# Peroral endoscopic myotomy for Zenker’s diverticulum without tunneling

**DOI:** 10.1055/a-2127-7402

**Published:** 2023-08-21

**Authors:** Georgios Mavrogenis, Fateh Bazerbachi

**Affiliations:** 1 Unit of Hybrid Interventional Endoscopy, Department of Gastroenterology, Mediterraneo Hospital, Athens, Greece; 2 CentraCare, Interventional Endoscopy Program, St. Cloud Hospital, St Cloud, Minnesota, USA

Peroral endoscopic myotomy (POEM) is an increasingly adopted strategy for the treatment of Zenker’s diverticulum ( Fig. 1 ). The Z-POEM technique allows for a deep incision of the cricopharyngeal muscle up to its transition to the esophageal muscle layer, and therefore more complete myotomy than direct diverticulotomy. Several modifications of the standard technique have been demonstrated 1 2 3 . The mucosal incision is most commonly performed over the septum, and two tunnels are created along the cricopharyngeal muscle. Next, the muscle is transected. Recently, we have described a variation of this technique with the creation of a single tunnel instead of two, to shorten the duration of the procedure 3 . However, we have now shifted to a novel approach that obviates the need for double or single tunneling ( Video 1 ).

**Fig. 1:**
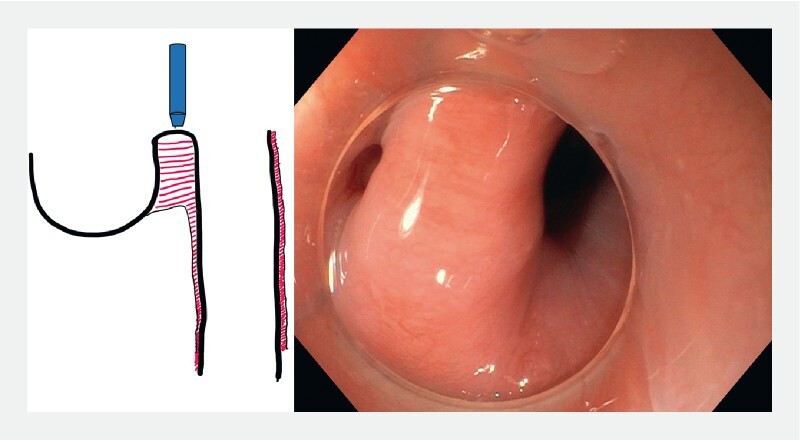
Scheme and endoscopic view of a typical Zenker’s diverticulum.

**Video 1** Peroral endoscopic myotomy for Zenker’s diverticulum without tunneling.


After the initial mucosal incision, both submucosal sides of the septum are lifted, with a mixture of hydroxyethyl starch and indigo carmine. Then we proceed to direct myotomy of the septum ( Fig. 2 ). The distended submucosal space on both sides of the septum functions as a protective cushion and prevents mucosal injury. After 1 cm of septotomy, further progression becomes much easier due to the increased space created by the partial myotomy ( Fig. 3 ). Further submucosal injections are provided as needed in order to maintain protective cushions on both sides. Myotomy is extended up to the proximal part, approximately 2 cm, of the esophageal muscle to reduce the risk of recurrence ( Fig. 4 ). Prior to endoscopic closure, it is important to examine for remaining intact muscle fibers at the proximal part of the septum ( Fig. 5 ). In our experience, this modification further shortens the duration of the procedure, with a median duration of 25 minutes, and is helpful where fibrosis is present from previous surgical interventions in the hypopharynx.

**Fig. 2:**
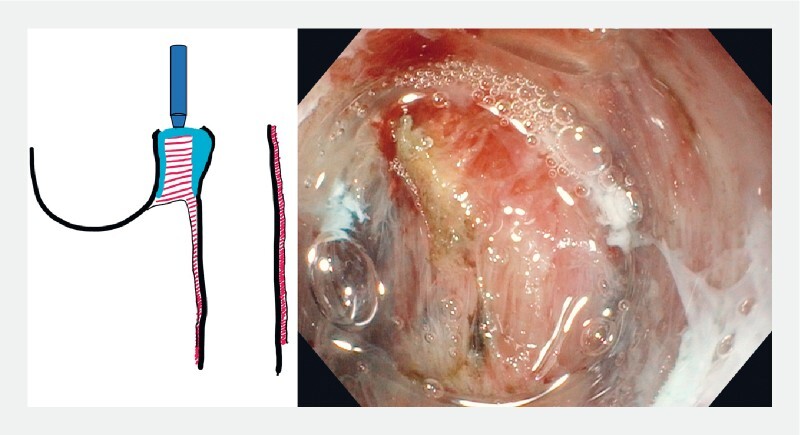
After mucosal incision, myotomy is performed. The submucosal space acts as a cushion protecting against mucosal injury.

**Fig. 3:**
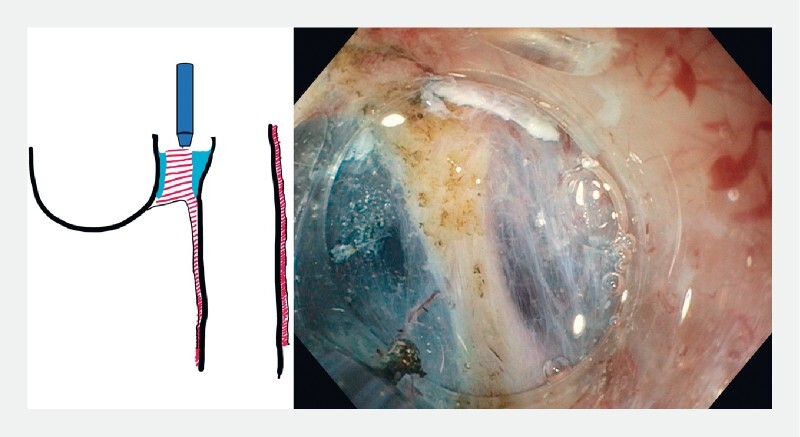
Myotomy without tunneling.

**Fig. 4:**
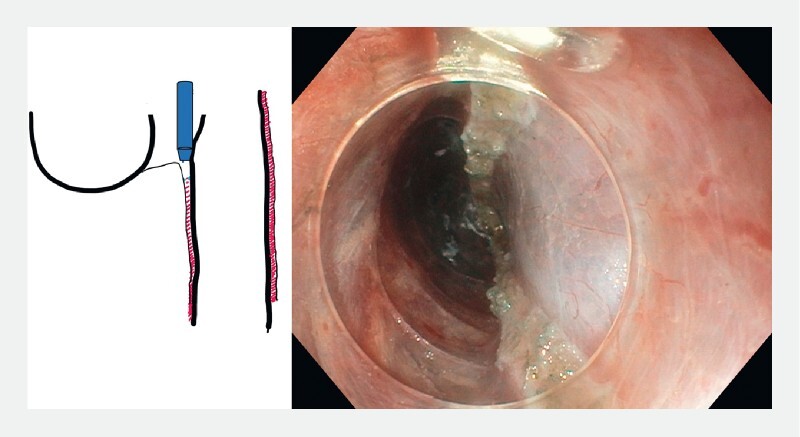
Myotomy extended up to the level of the upper esophagus.

**Fig. 5:**
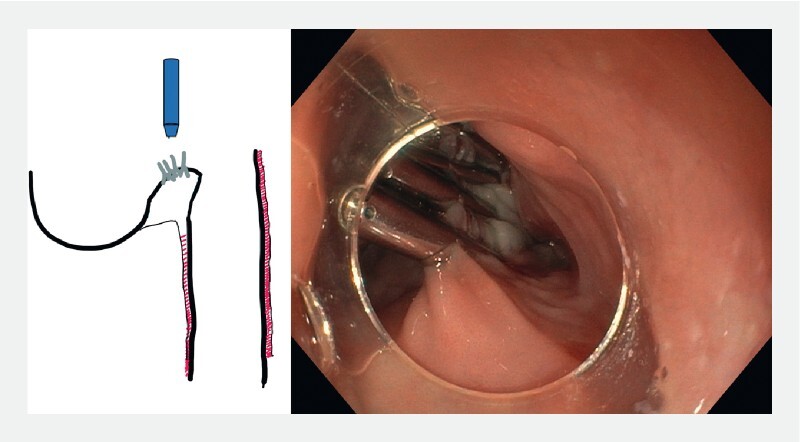
Closure of the mucosotomy.

Endoscopy_UCTN_Code_TTT_1AO_2AG

## Notes

Endoscopy E-Videos

https://eref.thieme.de/e-videos
*E-Videos* is an open access online section of the journal *Endoscopy* , reporting on interesting cases and new techniques in gastroenterological endoscopy. All papers include a high-quality video and are published with a Creative Commons CC-BY license. Endoscopy E-Videos qualify for HINARI discounts and waivers and eligibility is automatically checked during the submission process. We grant 100% waivers to articles whose corresponding authors are based in Group A countries and 50% waivers to those who are based in Group B countries as classified by Research4Life (see: https://www.research4life.org/access/eligibility/ ). This section has its own submission website at https://mc.manuscriptcentral.com/e-videos
